# The Chicken or the Egg? The Direction of the Relationship Between Mathematics Anxiety and Mathematics Performance

**DOI:** 10.3389/fpsyg.2015.01987

**Published:** 2016-01-07

**Authors:** Emma Carey, Francesca Hill, Amy Devine, Dénes Szücs

**Affiliations:** Centre for Neuroscience in Education, Department of Psychology, University of CambridgeCambridge, UK

**Keywords:** mathematics anxiety, mathematics performance, debilitating anxiety, deficit theory, cognitive interference, working memory, educational psychology

## Abstract

This review considers the two possible causal directions between mathematics anxiety (MA) and poor mathematics performance. Either poor maths performance may elicit MA (referred to as the Deficit Theory), or MA may reduce future maths performance (referred to as the Debilitating Anxiety Model). The evidence is in conflict: the Deficit Theory is supported by longitudinal studies and studies of children with mathematical learning disabilities, but the Debilitating Anxiety Model is supported by research which manipulates anxiety levels and observes a change in mathematics performance. It is suggested that this mixture of evidence might indicate a bidirectional relationship between MA and mathematics performance (the Reciprocal Theory), in which MA and mathematics performance can influence one another in a vicious cycle.

## Introduction

A pertinent question in mathematics anxiety (MA) research is whether MA causes poor maths performance, or whether poor maths performance elicits MA. This paper will review the extant literature to consider the possible models, and to provide greater insight into the nature of the MA-maths performance relationship.

Mathematics anxiety can be defined as a state of discomfort around the performance of mathematical tasks (Ma and Xu, 2004), and is generally measured using self-report trait anxiety questionnaires. There is broad consensus that MA is linked to poorer maths performance, with studies typically observing small to moderate negative correlations (Ashcraft and Krause, 2007; Devine et al., 2012; Zakaria et al., 2012; Jansen et al., 2013). For example, two meta-analyses found correlations of −0.27 and −0.34 between MA and maths performance (Hembree, 1990; Ma, 1999). Similar correlations have long been observed in non-maths specific anxiety, for example, between test anxiety and performance (Mandler and Sarason, 1952).

However, studies attempting to elucidate the direction of the MA-maths performance link are in conflict (Devine et al., 2012) and there is a paucity of longitudinal studies on the subject. Furthermore, the question is not trivial, since it should feed directly into educational policy. Beilock and Willingham (2014, p. 29) note that some believe “math(s) anxiety is just another name for ‘bad at math(s);”’ if policy-makers share this belief, to reduce students’ MA, effort and money will be targeted at courses to improve their maths. If the relationship is in fact in the other direction, such efforts are likely to be ineffective and it would be better to focus on alleviating MA to improve maths performance (Beilock and Willingham, 2014). On the other hand, if poor performance causes MA, it is possible that alternative teaching methods could mitigate this.

Knowing the direction of the MA-maths performance relationship has further implications for education and psychology research. For example, if poor performance is seen to increase MA, computer-adaptive programs may offer a way to ensure that students do not experience excessive failure in their maths learning, by adjusting the difficulty level to an individual student’s ability (as in Jansen et al., 2013). On the other hand, if MA reduces maths performance, further research is required into remediation of MA, particularly methods which may be undertaken in the maths classroom. For example, writing about emotions prior to a maths test has been seen to increase performance in those with high MA (Park et al., 2014).

The anxiety-performance link has two possible causal directions, which have been extended into the specific field of MA (Hembree, 1990). The first of these directions is encapsulated by the Deficit Theory, which claims that poor performance, for example in tests or maths, leads to higher anxiety about that situation in the future (Tobias, 1986). Proponents of the Deficit Theory believe that prior maths performance deficits lead to memories of poor maths performance, generating MA (Hembree, 1990).

The second causal direction is that anxiety reduces performance by affecting the pre-processing, processing, and retrieval of information (Wine, 1971; Tobias and Deutsch, 1980; Tobias, 1986), henceforth referred to as the Debilitating Anxiety Model. Prior to information processing, MA may influence learning by disposing individuals to avoid maths-related situations (Hembree, 1990; Chinn, 2009). Later, at the stages of processing and recall, MA may influence performance by cognitive interference. For example, MA may tax working memory resources, which are vital for the processing and retrieval of mathematical facts and methods (Ashcraft and Kirk, 2001; Ashcraft and Krause, 2007; Krinzinger et al., 2009). Indeed, research indicates that positive emotions enhance learning by increasing the persistence, strategy and recruitment of cognitive resources (Pekrun et al., 2002; Sabourin and Lester, 2014; Verkijika and De Wet, 2015) and that negative emotions, including anxiety, do the opposite (Meyer and Turner, 2006; Sabourin and Lester, 2014; Verkijika and De Wet, 2015). The multitude of studies indicating that emotions have an effect on general achievement supports the application of this theory to MA more specifically.

It is important to note that regardless of causal direction, other factors may well mediate or moderate the relationship between anxiety and performance. For example, academic self-concept has been identified as a factor related to academic performance (as in Guay et al., 2003), and low maths self-concept is related to MA (Ahmed et al., 2012). This mini-review focuses only on the direction of the relationship between MA and performance, rather than its many possible mediators and moderators.

Additionally, since deficit and debilitating anxiety theories may be applied to anxiety outside of the field of maths, we sometimes examine research into anxiety more generally. Whilst this forms a theoretical basis for deficit- and debilitating anxiety-based models, it is possible that MA and maths performance have a different causal relationship than do other forms of anxiety. Researchers have identified certain key beliefs held about maths (see Theoretical Review in Jackson, 2008 for a summary), which could moderate causal relationships, making MA different in nature from other forms of anxiety. Thus we focus on research on MA specifically, using research into other anxiety types only where similar research on MA is unavailable but may be useful to carry out.

## The Deficit Theory

Evidence revealing that children with mathematical learning disabilities are often found to have disproportionately high levels of MA, provides support for the Deficit Theory. It is likely that, in at least some cases, having especially poor maths performance in early childhood could elicit MA. In Italian fourth graders and Canadian 7–13 year-olds, those with mathematical learning disabilities display higher levels of MA than typically developing children (Rubinsten and Tannock, 2010; Passolunghi, 2011). However, whilst these studies of developmental dyscalculia and mathematical learning disabilities indicate that specific cases of MA are related to poor performance, with only 1–6% of the population suffering from developmental dyscalculia (Devine et al., 2013), such findings cannot straightforwardly be generalized to the typically developing child. It should also be noted that cognitive resources are not the only possible deficit which could cause poor maths performance and MA. For example, self-regulation deficits have been associated both with MA (Jain and Dowson, 2009; Kramarski et al., 2010) and decreased maths performance (Lee et al., 2014).

Longitudinal studies of typically developing children and adolescents also provide support for the Deficit Theory. One of few longitudinal studies in this area looked at adolescents in the United States, and found significant correlations (−0.11 to −0.2) between a student’s academic performance in one year and their MA in the following year (Ma and Xu, 2004). These correlations were stronger than those found between a student’s MA in one year and their academic performance in the following year, indicating that maths performance may cause MA, thus providing support for the Deficit Theory. Nevertheless, these results should be taken with caution. The mechanisms of influence proposed by the Debilitating Anxiety Model, particularly cognitive interference, may be more immediate than from one academic year to the next, since the effect of anxiety on recall would cause a fairly immediate performance decrement in those with high MA. If the Debilitating Anxiety Model were in operation, the effect of MA on performance may not be visible in MA-performance correlations from one year to the next. Thus whilst this research supports the idea that low maths performance may cause anxiety, it says nothing about whether there is also a relationship in the other direction.

In further support of the Deficit Theory, additional longitudinal research into MA in early adolescence similarly found that one year’s perceived maths ability was moderately correlated with the following year’s MA (Meece et al., 1990). However, MA was only measured in the second year of the 2-year study, and again MA and the same year’s performance were not compared, making comparison between the Deficit and Debilitating Anxiety models unfeasible.

Some researchers have suggested that MA in adults may result from a deficit in basic numerical processing, which would be more in line with the Deficit Theory. For instance, Maloney et al. (2010, 2011) have revealed that adults with high MA have numerical processing deficits compared to adults with low MA. The authors tentatively stated that the findings from these studies indicate that “MA may result from a basic low-level deficit in numerical processing that compromises the development of higher level mathematical skills” (Maloney et al., 2011, p. 14). However, as these studies did not follow the developmental trajectory of MA or the acquisition of mathematics skills in their participants, the authors could not determine the direction of the MA-maths performance relationship. Importantly, these results do not preclude the possibility that highly maths anxious adults’ basic numerical abilities were impaired because they have avoided mathematical tasks throughout their education and in adulthood due to their high levels of MA, which would be more consistent with the Debilitating Anxiety Model.

Genetic studies may help to elucidate whether maths performance deficits do in fact emerge first and cause MA to develop. One such study suggests that 9% of total variance in MA stems from genes related to general anxiety, and 12% from genes related to maths cognition (Wang et al., 2014). This may indicate that for some, MA is caused by a genetic predisposition to deficits in maths cognition. However, it does not preclude the possibility that the relationship between MA and performance is reciprocal. It may be useful to study those individuals who experience MA but do *not* have the genes associated with maths performance deficits, in order to see whether performance deficits can emerge from MA alone.

## The Debilitating Anxiety Model

Many alternative studies across childhood, adolescence, and adulthood provide support for the Debilitating Anxiety Model, suggesting that MA can impact performance at the stages of pre-processing, processing and retrieval of maths knowledge. Hembree’s (1990) meta-analysis included evidence suggesting that adolescents with MA may avoid maths-related situations, pointing to the idea that MA is likely to exert an influence on performance by reducing learning opportunities. Similarly, Ashcraft and Faust (1994) found that adults with high MA answered maths questions less accurately but more quickly than those with lower levels, and Morsanyi et al. (2014) found that MA was associated with decreased cognitive reflection during mathematics word problems. Such data suggest that adults with MA may avoid processing mathematical problems altogether which could lead both to reduced maths learning and to lower maths performance due to rushing. Further support comes from the wealth of evidence indicating that adults with MA are less likely to enroll on college or university courses involving mathematics (for a review see Hembree, 1990). Even in young children, task-avoidant behaviors have been found to reduce maths performance (Hirvonen et al., 2012). Furthermore, recent research suggests that anticipation of maths causes activation of the neural ‘pain network’ in high MA individuals, which may help to explain why high MA individuals are inclined to avoid maths (Lyons and Beilock, 2012b). This strongly suggests that MA is likely to influence adults’ maths outcomes at the pre-processing level, providing support for the Debilitating Anxiety Model.

Additionally, there is evidence that MA impairs maths performance during maths processing by taxing processing resources. Eysenck and Calvo’s (1992) Processing Efficiency Theory suggests that worry reduces working memory’s processing and storage capacity, thus reducing performance. Ashcraft and Kirk (2001) found a negative correlation between college students’ MA levels and their working memory span. Further, Ashcraft and Krause (2007) found an interaction between adults’ MA and their performance on high and low working-memory load maths problems, with high working-memory load questions being more affected by MA. Thus, MA appears to exert an effect on performance by compromising the working-memory functions of those with high MA. It is also possible that MA affects strategy selection, leading individuals to choose simpler and less effective problem-solving strategies and thus impairing their performance on questions with a high working-memory load (Beilock and Decaro, 2007). This is supported by evidence suggesting that those with high working-memory, who usually use working-memory intensive strategies, are more impaired under pressure than those who tend to use simpler strategies (Beilock and Carr, 2005; Ramirez et al., 2013).

Experimental studies attempt to solve the problem of the causal ordering of MA and maths deficits by manipulating MA only and observing whether this has an impact on performance. For example, it has been observed that engaging in free-writing about emotions prior to a maths test, in order to alleviate MA-related intrusive thoughts, increases performance (Park and Ramirez, 2014). Furthermore, MA is observed to be less linked to maths performance when maths tests are not timed, indicating that anxiety resulting from time-pressure reduces test performance (Faust et al., 1996). Both of these studies provide support for the cognitive interference proposed within the Debilitating Anxiety Model, since they highlight the negative effects MA can have on maths test performance.

Stereotype threat studies manipulate anxiety levels in the opposite direction, and also indicate that the Debilitating Anxiety Model may best explain the causal ordering of the MA and maths performance relationship. Stereotype threat is the situation in which members of a group are, or feel themselves to be, at risk of confirming a negative stereotype about their group. Under stereotype threat, individuals are seen to perform more poorly in a task than they do when not under this threat. It is posited that this is due to anxiety elicited by the potential to confirm or disconfirm a negative stereotype about one’s group (Steele and Aronson, 1995; Schmader et al., 2008).

Whilst not all studies of children and adolescents demonstrate the effect of stereotype threat on maths performance (see Ganley et al., 2013 for discussion), it appears that at least under some conditions, certain populations show an effect from stereotype threat based anxiety manipulations. For example, Galdi et al. (2013) found that Italian 6–7 year-old girls showed a performance decrement after completing a task to elicit stereotype threat prior to their maths assessment. The effect of increasing anxiety by stereotype threat can be seen in adults as well as children. For example, it has been observed that presenting women with a female role model who doubted her maths ability reduced their performance in maths problems compared with a control group who were presented with a non-doubtful female role model (Marx et al., 2013). This finding has been supported by other studies in adults (Spencer et al., 1999; Schmader, 2002; Gerstenberg et al., 2012; Seitchik et al., 2012). Deficits seen in women under maths stereotype threat appear to be mediated by a working-memory impairment, supporting the idea that MA influences performance by taxing working-memory resources (Beilock et al., 2007). Further, stereotype threat based maths performance decrements have been observed based on race and income level as well as gender (Tine and Gotlieb, 2013). Such data is in accordance with the Debilitating Anxiety Model, since anxiety manipulations demonstrate the deleterious effects of MA.

Neuroimaging data also suggest that the Debilitating Anxiety Model is in operation. Lyons and Beilock (2012a) carried out an fMRI study on high and low MA adults. Whilst there was a significant performance difference between high and low MA individuals, within-group correlations between MA and performance were not observed. This raises the question of how some individuals with very high MA outperform those with slightly lower, but still relatively high, MA. Neuroimaging revealed that in high MA individuals, increased activity in frontoparietal regions (involved in the cognitive control and reappraisal of negative emotions) prior to performing maths tasks was correlated with higher performance. This indicates that some high MA individuals are able to use higher cognitive functions to mitigate the effect of MA on performance, and may reveal why correlations between MA and performance tend to be relatively low, albeit significant. This is highly supportive of the Debilitating Anxiety Model: it appears that individuals who are better able to suppress their negative emotional response to maths have less of a performance deficit, and therefore suggests that the original performance deficit was caused by negative and intrusive thoughts (Lyons and Beilock, 2012a). A more recent fMRI study reached a similar conclusion after finding that MA did not affect activation in brain areas known to be involved in numerical processing (Pletzer et al., 2015). MA was instead linked with reduced deactivation of the Default Mode Network (see Pletzer et al., 2015 for details), indicating a preoccupation with the emotional value of numerical stimuli. This suggests that performance deficits in high MA individuals are more related to emotional interference than cognitive deficits.

## The Reciprocal Theory

The evidence is conflicting; some studies provide data which appears to fit the Deficit Theory, whereas others provide more support for the Debilitating Anxiety Model. However, there may be an explanation for such conflicting evidence. It may in fact be indicative of the very nature of the MA-maths performance relationship; whilst poor performance may trigger MA in certain individuals, it may further reduce their maths performance in a vicious cycle (as endorsed in Jansen et al., 2013). Ashcraft et al. (2007) propose a model in which MA can develop either from non-performance factors, such as biological predisposition, or from performance deficits. They argue MA may then cause further performance deficits, via avoidance and working-memory disruption, supporting the Reciprocal Theory. The question of whether the MA-maths performance relationship is in fact reciprocal is likely to be best answered by longitudinal studies across childhood and adolescence, since only longitudinal data can determine whether MA or weak performance is first to develop.

However, there is limited non-longitudinal data which already suggests that the Reciprocal Theory may provide the best explanation for the MA-maths performance relationship. For example, data collected in Singapore suggest that previous achievement may affect a student’s MA levels and that MA in turn affects future performance (Luo et al., 2014). Pekrun (2006) provides a putative reciprocal model in which control and value appraisals predict academic anxiety, which affects performance, and further proposes indirect feedback loops from performance to appraisals and emotions. In light of the conflicting evidence discussed, such complex models involving feedback loops between multiple factors, including MA and maths performance, are likely to provide the best explanation of the relationship between MA and maths performance.

Whilst researchers often provide data supporting either the Deficit Theory or the Debilitating Anxiety Model rather than endorsing a reciprocal model, it is possible that this relates to methodological constraints. In particular, the mechanisms proposed by the Deficit Theory are long-term, with the detrimental effect of poor performance on anxiety levels occurring over a number of years. This may be why the Deficit Theory is often supported by long-term longitudinal studies (e.g., Ma and Xu, 2004). On the other hand, the Debilitating Anxiety Model, particularly cognitive interference, proposes some immediate mechanisms for anxiety’s interference with performance (e.g., taxing working memory resources, as discussed in Ashcraft and Krause, 2007). This could explain why the Debilitating Anxiety Model is best supported by experimental studies such as those into stereotype threat. It is quite plausible that the limitations of carrying out just one study type (such as a long-term longitudinal investigation or a short-term experimental study) mean that studies reveal only one of two operational causal directions. Examining a variety of data, collected using different methods and over varied time scales, is likely to reveal whether methodological factors explain why the literature rarely supports the Reciprocal Theory.

To sum, the evidence relating to the relationship between MA and maths performance is mixed. There is research to support the Deficit Theory’s claim that poor past performance can cause MA, with the strongest evidence coming from longitudinal studies (Meece et al., 1990; Ma and Xu, 2004) and studies of mathematical learning disabilities (Rubinsten and Tannock, 2010; Passolunghi, 2011). Nevertheless, in support of the Debilitating Anxiety Model, there is evidence to suggest that anxiety can have a deleterious effect on maths performance. This is strongly supported by studies across all ages which manipulate anxiety to reveal either a decrement or improvement in performance. This effect of MA on performance is likely to be mediated by working-memory impairments caused by intrusive thoughts. However, neither theory can fully explain the relationship observed between MA and maths performance. The mixture of evidence may suggest a bidirectional relationship between MA and maths performance, in which poor performance can trigger MA in some individuals and MA can further reduce performance, in a vicious cycle. Nevertheless, more longitudinal and mixed-methods research is required to provide greater understanding into this relationship and more direct support for the Reciprocal Theory.

## Conflict of Interest Statement

The authors declare that the research was conducted in the absence of any commercial or financial relationships that could be construed as a potential conflict of interest.
